# Assessment of the Readability of Google Gemini-Generated Versus UpToDate Clinical Contents for Ventricular Tachycardia Management

**DOI:** 10.7759/cureus.97997

**Published:** 2025-11-28

**Authors:** Akash Ranganatha, Veena Ramanathan, Nikhil Vamsi Chitte, Mansi Chaudhari, Parv Panchal, Mohammad Danish

**Affiliations:** 1 Internal Medicine, Jagadguru Jayadeva Murugarajendra (JJM) Medical College, Davanagere, IND; 2 Internal Medicine, Davao Medical School Foundation, Inc., Davao City, PHL; 3 Internal Medicine, Kamineni Institute of Medical Sciences, Narketpally, IND; 4 Internal Medicine, Vibrant Multispeciality Hospital, Mehsana, IND; 5 Medicine, Dr. M. K. Shah Medical College and Research Centre, Ahmedabad, IND; 6 General Medicine, Swansea Bay University Health Board, Swansea, GBR

**Keywords:** artificial intelligence, cross-sectional study, evidence-based medicine, google gemini, readability, uptodate, ventricular tachycardia

## Abstract

Introduction

Ventricular tachycardia (VT) is a life-threatening arrhythmia requiring complex management. Google Gemini is increasingly used to generate medical content, while UpToDate remains a leading expert-authored clinical reference. This study compared the "readability," not the "clinical accuracy," of VT-related content generated by Google Gemini and UpToDate.

Aims

This study aims to objectively compare the readability of Google Gemini-generated medical content and UpToDate expert-authored material on VT using standardized readability indices.

Methods

A cross-sectional study analyzed 12 textual responses on VT management: six from Google Gemini and six from UpToDate. Readability was assessed using the WebFX Readability Test Tool, which calculates Flesch Reading Ease (FRE), Flesch-Kincaid Grade Level (FKGL), Simple Measure of Gobbledygook (SMOG) Index, and Difficult Word Percentage. As the data were non-normally distributed, the Mann-Whitney U test was applied. The analysis was limited to quantitative readability metrics and did not assess clinical accuracy or human comprehension.

Results

The median FKGL score was 16.5 for UpToDate and 13.5 for Google Gemini (p = 0.017), indicating that UpToDate text required a more advanced reading level. The median Difficult Word Percentage was 32.0% for Google Gemini and 28.6% for UpToDate (p = 0.026), suggesting that Google Gemini content contained a higher proportion of multisyllabic or specialized words. No significant differences were observed in FRE or SMOG Index values (p > 0.05).

Conclusion

Google Gemini-generated content demonstrated a simpler sentence structure but greater lexical density compared to UpToDate. This highlights a distinct linguistic pattern: syntactic simplicity combined with concentrated technical vocabulary. These findings emphasize the importance of expert review when using Google Gemini-generated material in medical education and communication.

## Introduction

Ventricular tachycardia (VT) is a life-threatening cardiac arrhythmia characterized by a rapid rhythm originating from the ventricles. It can occur in structurally normal or diseased hearts and is often associated with ischemic cardiomyopathy, myocardial infarction, or other cardiomyopathies. Sustained VT, if untreated, may degenerate into ventricular fibrillation, leading to sudden cardiac arrest and death. Clinicians frequently encounter presentations ranging from benign forms to those with significant risk of fatal arrhythmic events [1]. Management remains complex, requiring pharmacological therapy, implantable cardioverter-defibrillators (ICDs), catheter ablation, and long-term risk stratification.

Recent advancements in artificial intelligence (AI) have introduced new approaches to arrhythmia management by improving diagnostic precision, treatment selection, and patient-specific risk prediction [2,3]. Traditional evidence-based methods, although validated, often rely on static clinical parameters, such as left ventricular ejection fraction, that may not capture dynamic risk variation over time [2]. AI systems, including machine learning and deep learning algorithms, enable rapid data analysis and pattern recognition across large datasets, enhancing both diagnostic and prognostic accuracy [4]. Studies have demonstrated diagnostic accuracies exceeding 80% in arrhythmia interpretation using AI-assisted electrocardiogram (ECG) analysis [3,5,6].

Multimodal AI frameworks such as DEEP RISK have improved identification of high-risk non-ischemic cardiomyopathy patients, achieving predictive performance with an area under the curve (AUC) approaching 0.84 [7]. Machine learning approaches that analyze sequential ECG data over time have significantly outperformed conventional static risk models in predicting malignant ventricular arrhythmias [8]. Furthermore, an international consensus statement from major cardiology societies, including the European Heart Rhythm Association (EHRA) and the Heart Rhythm Society (HRS), underscores the growing role of AI in clinical electrophysiology, encompassing arrhythmia detection, risk prediction, and procedural planning, and provides guidance for safe integration into clinical practice [9]. In addition, machine-learning-derived metrics of cycle-length variability from ICD data have been shown to help identify VT episodes likely to terminate spontaneously, thereby supporting more tailored therapy decisions [10].

These advances illustrate AI's expanding influence in cardiology and its potential to complement established evidence-based resources. However, as AI-generated medical content becomes increasingly accessible, questions arise about its readability and comprehensibility compared to traditional expert-authored references. Readability, the ease with which written text can be understood, plays a key role in ensuring effective learning and information transfer among medical professionals [11-13]. Tools such as the Flesch Reading Ease (FRE), Flesch-Kincaid Grade Level (FKGL), and Simple Measure of Gobbledygook (SMOG) index are widely used to quantify textual complexity and have been validated in multiple health communication studies [12,13].

Given the rapid adoption of AI tools in generating educational and clinical content, assessing their readability is crucial to understanding whether they enhance or hinder medical comprehension. This study aims to compare the readability of VT-related medical content generated by Google Gemini (AI) and UpToDate (expert-authored) to determine which source presents information in a more accessible format for healthcare readers. By focusing exclusively on objective readability indices, this research provides a quantitative foundation for understanding how AI-generated clinical writing aligns with established medical reference standards.

## Materials and methods

Study design and overview

This cross-sectional, AI-assisted educational study was conducted in July 2025 to compare the readability of medical content generated by Google Gemini and UpToDate. The study focused exclusively on quantitative readability assessment. No evaluation of clinical accuracy, completeness, or therapeutic validity was performed. Since the study involved only secondary, publicly available, non-identifiable textual data, institutional ethics approval was not required, and it qualified as non-human participant research under the U.S. Department of Health and Human Services (45 CFR 46).

Data sources and topic selection

Textual content was obtained from two sources: AI-generated material from Google Gemini and expert-authored material from UpToDate. Six representative clinical topics on VT were selected directly from UpToDate to ensure thematic consistency and clinical relevance. The topics were sustained monomorphic VT, wide QRS complex tachycardia, acute management of tachyarrhythmias, ventricular arrhythmia during acute myocardial infarction, non-sustained VT, and wide complex tachycardia in children. Each topic title was used as a standardized prompt in Google Gemini using the query "Write a detailed educational guide for medical professionals on [insert topic]." This design ensured identical subject representation across both sources while maintaining independence of content generation.

The sample size of six topics was chosen to cover distinct subtypes and management contexts for VT while maintaining methodological feasibility for detailed readability analysis. The selection aimed for conceptual breadth rather than statistical generalizability.

Data collection and review process

Content from UpToDate was accessed, and corresponding AI-generated text was produced contemporaneously in July 2025 to minimize version bias. All text was converted into plain text format and reviewed by two independent authors to ensure that only the main body content (excluding references, tables, and hyperlinks) was included for analysis. No manual modifications were made to either AI-generated or UpToDate text beyond formatting standardization. The reviewers' role was limited to ensuring consistency in text formatting and completeness for analysis; they did not edit or evaluate the accuracy or content validity of the material.

Readability assessment and metrics

The readability of each text sample was assessed using the WebFX Readability Test Tool, which calculates multiple standardized readability indices. The following measures were extracted: (i) FRE, which reflects ease of comprehension; higher scores indicate easier readability; (ii) FKGL, which indicates the U.S. grade level required for comprehension; higher values denote greater complexity; (iii) SMOG Index, which estimates the years of education required to understand the text; and (iv) the Difficult Word Percentage, which represents the proportion of words containing three or more syllables, calculated as (number of difficult words ÷ total word count) × 100. This measure served as an indicator of lexical difficulty [12,13]. All readability measures, including percentages, were treated as continuous variables, consistent with prior readability research conventions. The metrics were analyzed as independent text-level observations for each group.

Statistical analysis

All statistical analyses were performed using IBM SPSS Statistics for Windows, Version 25 (Released 2017; IBM Corp., Armonk, New York) and R Version 4.3.2 (The R Foundation, Vienna, Austria). The normality of distributions was evaluated using both visual inspection (histograms) and the Kolmogorov-Smirnov test. As all variables were non-normally distributed, non-parametric methods were applied.

Comparisons between Google Gemini and UpToDate were conducted using the Mann-Whitney U test, which was deemed appropriate because the text samples were independently generated and not directly paired despite topic-level correspondence. The level of statistical significance was set at α = 0.05.

Given the small sample size (six per group), the results are interpreted as exploratory, acknowledging limited statistical power and generalizability.

## Results

A total of 12 textual responses, six each from Google Gemini and UpToDate, were analyzed across six standardized topics on VT management: sustained monomorphic VT, wide QRS complex tachycardia, acute management of tachyarrhythmias, ventricular arrhythmia during acute myocardial infarction, non-sustained VT, and wide complex tachycardia in children. Each topic pair was evaluated using four continuous readability indices: FRE, FKGL, SMOG Index, and Difficult Word Percentage.

All readability variables were treated as continuous measures. Data distribution was non-normal, so non-parametric Mann-Whitney U tests were used for comparisons between the two groups. No categorical variables were analyzed.

Overall readability comparison

UpToDate content consistently demonstrated higher textual complexity and length across most parameters. The median FKGL score was significantly higher for UpToDate than for Google Gemini (16.5 vs. 13.5; p = 0.017), indicating that UpToDate text required a more advanced reading level. In contrast, Google Gemini content exhibited a significantly higher Difficult Word Percentage (32.0% vs. 28.6%; p = 0.026), suggesting that while sentence structure was simpler, the vocabulary was more specialized or technical. No statistically significant differences were noted for the FRE or SMOG Index scores (p > 0.05).

This pattern reflects a distinct readability profile: AI-generated text (Google Gemini) is syntactically simpler but lexically denser, whereas UpToDate content is more verbose and academically complex. This finding represents the study's key outcome and is discussed further in the next section in terms of educational implications.

Table 1 summarizes the readability indices used and the rationale for their classification. The FRE and FKGL measure sentence and word complexity, while SMOG estimates the educational level required for comprehension. Difficult Word Percentage identifies the proportion of multisyllabic words, serving as a proxy for lexical difficulty. The table outlines thresholds for each metric (e.g., FRE < 30 = difficult, FKGL ≥ 14 = academic, SMOG ≥ 13 = complex), clarifying how each measure contributes to the evaluation of text difficulty. This provides a standardized framework for interpreting the comparative results in Table 2.

**Table 1:** Readability metrics assessed and classifications

Readability Metric	Data Type	Classification Rationale
Word Count	Continuous	High/Low (based on median split or quartiles)
Sentence Count	Continuous	High/Low (varies based on source length and structure)
Words per Sentence	Continuous	>20 = complex; ≤20 = simple
Flesch Reading Ease (FRE)	Continuous	<30 = difficult; 30–59 = fairly difficult; ≥60 = easy
FK Grade Level (FKGL)	Continuous	<14 = easier; ≥14 = academic/complex
SMOG Index	Continuous	<13 = more accessible; ≥13 = more complex
Difficult Word Count	Continuous	High/Low (based on distribution)
Difficult Word Percentage	Continuous	≤30% = standard; >30% = high complexity

**Table 2:** Characteristics of responses generated by UpToDate and Google Gemini ^+^Mann-Whitney U test. *P-values < 0.05 are considered statistically significant.

Variable	Median (IQR)		U Statistic	P-value^+^
	UpToDate	Google Gemini		
Word Count	4593.0 (2942.5–6008.2)	1677.0 (1324.8–2046.5)	6	0.065
Sentence Count	227.5 (135.0–351.8)	138.0 (110.8–175.0)	7	0.093
Word/Sentence Count	22.5 (17.8–24.3)	12.3 (11.3–13.1)	6	0.065
Flesch Reading Ease (FRE)	16.9 (11.9–19.8)	19.2 (5.3–24.9)	15.5	0.732
FK Grade Level (FKGL)	16.5 (15.4–17.2)	13.5 (12.7–15.9)	3.5	0.017*
SMOG Index	14.5 (13.1–15.1)	11.4 (10.5–12.5)	6	0.056
Difficult Word Count	1351.5 (817.0–1683.5)	554.0 (422.8–645.2)	6	0.065
Difficult Word Percentage	28.6 (27.6–29.3)	32.0 (29.6–35.2)	4	0.026*

Table 2 presents the comparative readability statistics between Google Gemini and UpToDate across all indices. It summarizes the distribution of word count, sentence count, words per sentence, FRE, FKGL, SMOG Index, difficult word count, and difficult word percentage for both sources, reported as medians with interquartile ranges (IQRs). UpToDate showed higher median word count (4593.0 vs. 1677.0) and sentence count (227.5 vs. 138.0), reflecting longer and more detailed content. The median words per sentence were also greater in UpToDate (22.5 vs. 12.3), indicating higher syntactic complexity. However, the difference was statistically significant only for FKGL and Difficult Word Percentage, with p-values of 0.017 and 0.026, respectively. The table clearly illustrates that although Google Gemini text appeared easier to read due to shorter sentences and lower grade-level scores, it contained a greater proportion of multisyllabic or specialized words, reflecting a denser technical lexicon. No statistically significant differences were observed for FRE or SMOG Index values, suggesting that general readability formulas might not fully capture the nuanced balance between structure and vocabulary in AI-generated medical writing.

**Figure 1:**
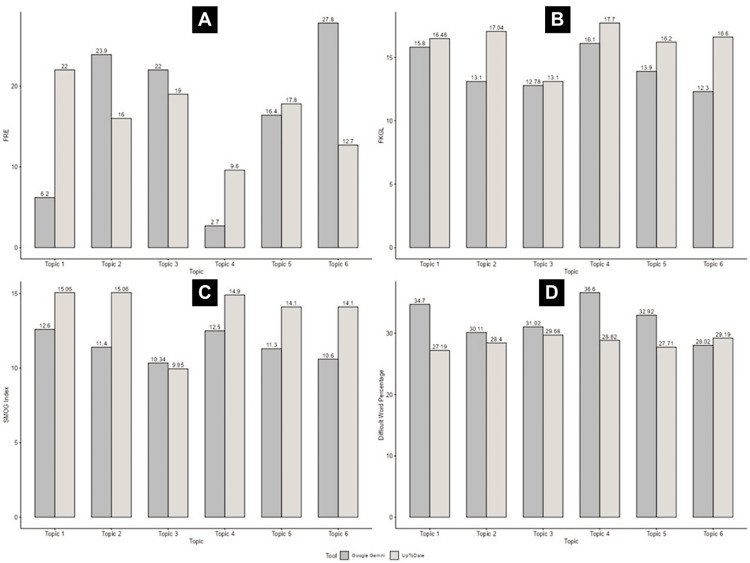
Graphical comparison of readability metrics between Google Gemini and UpToDate content on ventricular tachycardia management across six clinical topics (A) FRE = Flesch Reading Ease; (B) FKGL = Flesch-Kincaid Grade Level; (C) SMOG Index = Simple Measure of Gobbledygook Index; (D) Difficult Word % = Percentage of difficult words in the text. Gray bars represent Google Gemini content, and beige bars represent UpToDate content. This layout allows direct visualization of readability variations across topics without requiring reference to the main text. Topic 1 is Sustained Monomorphic Ventricular Tachycardia. Topic 2 represents Wide QRS Complex Tachycardia. Topic 3 is Acute Management of Tachyarrhythmias. Topic 4 represents Ventricular Arrhythmia During Acute Myocardial Infarction. Topic 5 is Non-Sustained Ventricular Tachycardia. Topic 6 represents Wide Complex Tachycardia in Children.

Figure 1 provides a graphical comparison of these readability metrics across the six clinical topics. Each bar corresponds to the raw numerical values rather than aggregated means or standard deviations, allowing visualization of individual topic variability. The upper left panel represents FRE, the upper right panel depicts FKGL, the lower left panel displays the SMOG Index, and the lower right panel shows the Difficult Word Percentage. Gray bars denote Google Gemini, and beige bars denote UpToDate content. The figure highlights how UpToDate text consistently required a higher reading level (higher FKGL and SMOG Index) and contained longer sentences, whereas Google Gemini text maintained a shorter structure but a higher proportion of difficult words. The visible pattern across panels supports the statistical findings, showing that AI-generated text tends to simplify syntax but compresses complex terminology into shorter statements, while UpToDate spreads complex ideas across longer sentences with greater explanatory detail.

Overall, the results indicate that Google Gemini content was shorter and structurally more straightforward, using simpler grammar and fewer words per sentence. However, it was also more jargon-heavy, containing a higher proportion of complex, multisyllabic terms. This contrast underlines the paradoxical nature of AI-generated medical content; it appears more readable by formulaic standards yet may be harder to understand for readers unfamiliar with technical language. The following section further discusses the educational implications of this finding.

## Discussion

This study compared the readability of medical text on VT generated by Google Gemini with expert-authored material from UpToDate. Across six standardized topics, Google Gemini text demonstrated lower syntactic complexity, reflected by a lower FKGL score (median 13.5 vs. 16.5; p = 0.017), while UpToDate text was significantly longer, with a higher median word count (4593 vs. 1677) and sentence count (227.5 vs. 138). In contrast, Google Gemini content exhibited a higher Difficult Word Percentage (32.0% vs. 28.6%; p = 0.026), despite its simpler sentence structure. No significant differences were noted in FRE or SMOG Index values (p > 0.05), suggesting comparable overall difficulty levels [12,13]. Together, these findings reveal a consistent pattern: AI-generated content is structurally simpler but lexically denser, a phenomenon described here as "jargon-packing."

The "jargon-packing" effect highlights a paradoxical feature of AI-generated medical content: its text may appear easier to read according to traditional readability formulas, yet it uses more technical terminology compressed into fewer words. This suggests that large language models prioritize concise phrasing while maintaining specialized terminology to preserve perceived authority. Previous studies comparing AI tools such as ChatGPT with traditional educational materials have similarly noted that while AI text often meets recommended readability levels, it may oversimplify or misrepresent complex medical nuances [13-15]. In contrast, traditional expert-authored sources such as UpToDate, which showed a higher FKGL score in this study, maintain a structured and explanatory writing style that supports deeper comprehension but demands greater reading effort [16].

The educational implications of this readability-jargon paradox are significant. For medical learners or early trainees, Google Gemini's lower grade level (13.5) may facilitate quicker reading and initial understanding of key concepts. However, the higher proportion of difficult or multisyllabic words (32.0%) may challenge readers unfamiliar with advanced medical vocabulary, potentially leading to superficial comprehension. Conversely, UpToDate's content, though more complex in structure, provides detailed contextual framing that supports accurate interpretation [17-19]. Thus, AI-generated material may complement, but not replace, expert-reviewed resources; it can aid rapid access to information but still requires human oversight for conceptual clarity and reliability [20,21].

This study also offers insight into how readability formulas may inadequately capture interpretive complexity. The absence of significant differences in FRE and SMOG Index values (p > 0.05) indicates that sentence-level and syllable-based metrics do not fully account for the influence of specialized terminology [12,13]. Similar limitations have been identified in previous analyses, in which readability metrics failed to reflect true cognitive load or conceptual difficulty [17-19]. These findings reinforce the need for developing advanced readability tools tailored to medical communication, capable of integrating semantic and contextual factors alongside linguistic simplicity, a gap underscored by the "jargon-packing" phenomenon observed here.

Our findings are consistent with prior research showing improved surface readability but variable factual precision and interpretive reliability in AI-generated medical text [16-21]. While this study did not assess factual accuracy or content validity, earlier investigations have revealed that AI tools can generate understandable but sometimes incomplete or inaccurate medical content [22-24]. Nevertheless, readability assessment alone proved valuable in identifying distinctive linguistic trends in AI outputs and establishing quantitative evidence of contrasting writing patterns between AI and expert-authored sources.

Several limitations must be acknowledged. The small sample size (six per group) limits statistical power and generalizability. The analysis was cross-sectional, reflecting Google Gemini's and UpToDate's outputs as of July 2025; future updates to AI models may alter readability profiles. Additionally, traditional readability indices (FRE, FKGL, SMOG) focus on structural metrics such as word length and sentence complexity rather than conceptual accuracy or comprehension [12,13]. Despite these constraints, the consistency of findings across six diverse VT topics supports the internal validity of the results.

In summary, this study identifies a distinct linguistic pattern in AI-generated medical writing: simpler in structure but richer in jargon. The lower FKGL and shorter sentence length of Google Gemini indicate improved surface readability, but the higher proportion of difficult words reflects denser technical vocabulary. Recognizing and quantifying this "jargon-packing" effect provides a foundation for refining AI-based educational tools. As AI continues to influence how medical information is produced and consumed, future studies should integrate readability, factual accuracy, and human comprehension analyses to ensure that increased accessibility does not come at the cost of understanding [23,24].

## Conclusions

The findings highlight an emerging paradox in AI-generated medical writing: text that appears easier to read often conceals a dense concentration of technical language. This "jargon-packing" pattern suggests that surface readability alone does not guarantee genuine comprehension. In medical education and clinical communication, this duality carries important implications: clarity must coexist with precision, and accessibility must not come at the cost of understanding. AI can streamline knowledge sharing and support rapid access to complex information, but it cannot yet replace the depth, contextual framing, and interpretive accuracy of expert-authored resources. Meaningful integration of AI in medical education will therefore depend on combining algorithmic efficiency with human oversight, ensuring that generated content remains both readable and reliable.

Future research should explore how readers perceive and interpret AI-produced material across specialties, develop advanced readability frameworks tailored to medical language, and evaluate how terminology density affects learning and clinical decision-making. Recognizing and refining this balance between simplicity and substance will be essential to harnessing AI responsibly in healthcare communication and education.
